# Reference Genomes from Distantly Related Species Can Be Used for Discovery of Single Nucleotide Polymorphisms to Inform Conservation Management

**DOI:** 10.3390/genes10010009

**Published:** 2018-12-22

**Authors:** Stephanie J. Galla, Natalie J. Forsdick, Liz Brown, Marc P. Hoeppner, Michael Knapp, Richard F. Maloney, Roger Moraga, Anna W. Santure, Tammy E. Steeves

**Affiliations:** 1 School of Biological Sciences, University of Canterbury, Christchurch 8140, New Zealand; tammy.steeves@canterbury.ac.nz; 2 Department of Anatomy, University of Otago, Dunedin 9054, New Zealand; michael.knapp@otago.ac.nz; 3 Te Manahuna, Department of Conservation, Twizel 7901, New Zealand; lbrown@doc.govt.nz; 4 Institute of Clinical Molecular Biology, Kiel University, 24105 Kiel, Germany; m.hoeppner@ikmb.uni-kiel.de; 5 Science and Policy Group, Department of Conservation, Christchurch 8011, New Zealand; rmaloney@doc.govt.nz; 6 Tea Break Bioinformatics Ltd., Palmerston North 4144, New Zealand; r.moraga@protonmail.com; 7 School of Biological Sciences, University of Auckland, Auckland 1142, New Zealand; a.santure@auckland.ac.nz

**Keywords:** conservation genomics, conservation genomics gap, SNP discovery, B10K, threatened species, birds

## Abstract

Threatened species recovery programmes benefit from incorporating genomic data into conservation management strategies to enhance species recovery. However, a lack of readily available genomic resources, including conspecific reference genomes, often limits the inclusion of genomic data. Here, we investigate the utility of closely related high-quality reference genomes for single nucleotide polymorphism (SNP) discovery using the critically endangered kakī/black stilt (*Himantopus novaezelandiae*) and four Charadriiform reference genomes as proof of concept. We compare diversity estimates (i.e., nucleotide diversity, individual heterozygosity, and relatedness) based on kakī SNPs discovered from genotyping-by-sequencing and whole genome resequencing reads mapped to conordinal (killdeer, *Charadrius vociferus*), confamilial (pied avocet, *Recurvirostra avosetta*), congeneric (pied stilt, *Himantopus himantopus*) and conspecific reference genomes. Results indicate that diversity estimates calculated from SNPs discovered using closely related reference genomes correlate significantly with estimates calculated from SNPs discovered using a conspecific genome. Congeneric and confamilial references provide higher correlations and more similar measures of nucleotide diversity, individual heterozygosity, and relatedness. While conspecific genomes may be necessary to address other questions in conservation, SNP discovery using high-quality reference genomes of closely related species is a cost-effective approach for estimating diversity measures in threatened species.

## 1. Introduction

The field of conservation genetics is in transition from using relatively few genetic markers (e.g., microsatellites, mitochondrial sequences) to using thousands of genome-wide single nucleotide polymorphisms (SNPs) discovered with high-throughput sequencing technologies (HTS) to inform conservation management of threatened species. In addition to providing greater resolution for diversity estimates (e.g., nucleotide diversity, heterozygosity, relatedness [1]), these new genomic approaches provide an opportunity to tackle new questions regarding regions of the genome that underlie fitness-related traits (i.e., adaptive variation [2,3,4]). While the promise of a conservation genomic approach has been heralded for well over a decade [5], the uptake of these technologies by conservation management has been slow [6,7].

This time lag between technology availability and implementation (also termed the ‘conservation genomics gap’ [7]) may be caused by several interconnected issues, including a disconnect between conservation genetic researchers and practitioners [8,9], the time it takes for geneticists to upskill in bioinformatic expertise [6,7,10], and initial expense for HTS sequence production and generation of genomic resources (e.g., a high-quality reference genome). With that said, sequencing costs are dropping precipitously [11] (but see also [12]) and affordable reduced representation genomic approaches provide the ability to produce high-density marker sets, even in the absence of a reference genome (i.e., de novo marker discovery [13]). While it is possible to discover SNPs de novo, reference-guided approaches to SNP discovery offer many advantages, including enhanced computational efficiency, improved accuracy at low sequencing depth, higher confidence in identifying sequence contamination, greater ability to identify the location of SNPs, improved performance in determining linkage disequilibrium between SNPs, and greater ability to identify differences between paralogous and repetitive sequences from true SNP variants [14,15,16,17]. Reference genomes also allow for identifying variants in annotated gene regions, which is necessary for identifying adaptive variation [14]. While reference genomes are preferred for conservation genomic research, they are often unavailable for threatened species or out of reach for resource-constrained conservation projects (e.g., [18]).

There has been an exponential increase in the number of available eukaryotic genomes for non-model species that may be used as a reference [19], including the outputs from various genome consortiums (e.g., Genome 10K [20]; Bird 10,000 Genomes Project (B10K) [21]; 5000 Insect Genome Project (i5K) [22]; 1000 Plants Project (1KP) [23]; Oz Mammalian Genomics [24]; Earth BioGenome Project [25]). Readily available conspecific reference genomes for threatened species will likely enable faster uptake of a conservation genomics approach, for example, by avoiding the time and expenditure of sequencing and assembling a high-quality genome de novo. However, in many instances, the next best available resource may be a genome from a closely related species. There has been discussion on the utility of closely related reference genomes for reference-guided genome assembly (i.e., [26,27]). Additionally, there are many research studies to date that have used closely related reference genomes for SNP discovery using reduced-representation and whole genome resequencing (hereafter, resequencing) approaches (e.g., [28,29,30,31]).

Birds offer an exceptional opportunity to study the utility of SNP discovery using closely related reference genomes to inform conservation management. In comparison with other vertebrates, bird genomes are relatively small (~0.93–1.3 Gb), compact (i.e., low repetitive elements), and conserved between species [32,33]. Also, the availability of bird reference genomes has increased, due in part to the efforts of individual research groups that produce genomes to answer questions regarding primary production (e.g., chicken, *Gallus gallus* [34]; the turkey, *Meleagris gallopavo* [35]), evolution (e.g., zebra finch, *Taeniopygia guttata*, [36]; Galapagos cormorant, *Phalacrocorax harrisi* [37]), and conservation (e.g., ‘amakihi/Hawaiian honeycreeper, *Hemignathus virens* [38]; ‘alalā/Hawaiian crow, *Corvus hawaiiensis* [39]; kākāpō, *Strigops habroptilus* [40]; kakī/black stilt, *Himantopus novaezelandiae*, this study). A substantial increase in the number of reference genomes available for birds can also be attributed to the efforts of B10K [21,41], the international consortium whose goal is to produce a genome for every known species of bird. To date, B10K has published 38 de novo bird reference genomes [21]. These genomes, along with others that were available at the time of publication, make genomic resources available for at least one individual in almost every order of class Aves [42]. The next phase of B10K will include genomes representing one species from every bird family (*n* = 240, [42]), increasing the availability of conspecific or closely related reference genomes for conservation research.

Here, we explore the utility of closely related reference genomes for SNP discovery using a critically endangered wading bird, the kakī, as proof of concept. Once found on the North and South Islands of New Zealand, kakī experienced significant population decline throughout the 20th century due to habitat loss and degradation, and the introduction of mammalian predators. Today, there are approximately 132 kakī remaining (New Zealand Department of Conservation, *unpublished data*) and the population is contingent upon intensive management [43,44], including a captive breeding and rearing programme that uses genetic-based estimates of relatedness to pair distantly related individuals in captivity [45]. Beyond kakī, many programmes for threatened species incorporate neutral genetic measures (e.g., nucleotide diversity, individual heterozygosity or inbreeding, and relatedness) into management plans to minimise inbreeding [46] and loss of diversity [47,48] to reduce extinction risk [49,50].

**Figure 1:**
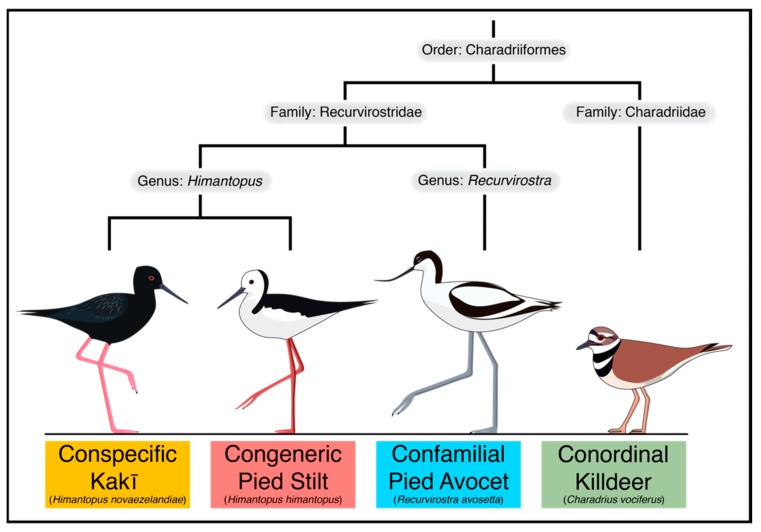
Evolutionary relationships between species with reference genomes used in this proof of concept. The evolutionary tree indicates topology between taxa within the order Charadriiformes. Evolutionary tree is not to scale.

To demonstrate that closely related reference genomes can yield sufficient SNPs to estimate diversity measures in threatened species, we map kakī genotyping-by-sequencing (GBS) and resequencing reads to genomes from members across the order Charadriiformes, including a conspecific reference genome (kakī, family: Recurvirostridae, *H. novaezelandiae*), and members of the same genus (pied stilt, family: Recurvirostridae, *H. himantopus*), family (pied avocet, family: Recurvirostridae, *Recurvirostra avosetta*), and order (killdeer, family: Charadriidae, *Charadrius vociferus*) (Figure 1). Members from this comparison represent a wide evolutionary time scale: estimates based on traditional single-locus phylogenetic approaches suggest Charadriidae and Recurvirostridae diverged approximately 69 million years ago, avocets (genus: *Recurvirostra*) and stilts (genus: *Himantopus*) diverged approximately 36.9 million years ago, and kakī and pied stilt diverged approximately 1 million years ago [51,52] (but see [53]) (Figure 1). SNPs discovered from these reference-guided assemblies were then compared using estimates of diversity relevant to the conservation management of threatened species, including nucleotide diversity, individual heterozygosity, and relatedness.

## 2. Materials and Methods

### 2.1. Tissue Sampling and DNA Extractions

Kakī blood samples were collected during routine health checks by the New Zealand Department of Conservation (DOC) at the captive breeding facilities in Twizel (DOC) and Christchurch (Isaac Conservation and Wildlife Trust), New Zealand, by approval of the DOC Animal Ethics Committee (AEC #283). These samples were stored in 95% molecular grade ethanol at −80 °C at the University of Canterbury. Pied stilt blood samples were collected from one female and one male during routine health checks at Adelaide Zoo, with samples provided under the Royal Zoological Society of South Australia Specimen Licence Agreement (Import Permit: 2016061954). Pied stilt samples were stored in EDTA at −20 °C at the University of Otago. The pied avocet blood sample was collected from a single individual from Hamburger Hallig, Germany, under a permit from the Ministry of Energy, Agriculture, the Environment, Nature and Digitization of the federal state of Schleswig-Holstein, Germany (Permit: V312-7224.121-37 [42-3/13]). Pied avocet samples were stored on filter paper at −20 °C at the University of Kiel.

Genomic DNA for kakī and pied stilt reference genomes was extracted at the University of Otago using a Thermo Scientific™ MagJET™ Genomic DNA Kit (Waltham, USA) following manufacturer specifications. DNA was isolated for the pied avocet sample at the University of Kiel Institute for Clinical Molecular Biology (hereafter, IKMB) by adding 400 µL of phosphorus buffered saline solution (PBS) to dried blood and using the Qiagen^®^ QIAmp^®^ DNA Blood Mini QIAcube^®^ Kit (Hilden, Germany) following the manufacturer specifications. Genomic DNA for the kakī genotyping-by-sequencing (GBS) and resequencing approaches was extracted at the University of Canterbury using a lithium chloride chloroform extraction method (see Supplement 1 for details). Genomic DNA for all extractions were analysed for quality using a NanoDrop™ Spectrophotometer and for quantity using an Invitrogen™ Qubit™ Fluorometer.

### 2.2. Reference Genome Library Preparation and Sequencing

Paired-end libraries for the kakī and pied stilt were prepared at the University of Otago using the Illumina TruSeq^®^ DNA PCR-free protocol according to manufacturer specifications, with genomic DNA fragmented to 350 bp. End repair and adapter ligation for sequence barcoding were carried out and libraries were indexed with unique 6 bp sequences. Sequencing of kakī and pied stilt libraries was completed by New Zealand Genomics Limited (NZGL), where sample libraries were pooled with three additional stilt samples and spread across five lanes of a flow cell for 2 × 125 bp sequencing on an Illumina HiSeq 2500.

Paired-end libraries for the pied avocet were prepared using the TruSeq^®^ DNA Nano Library Prep protocol according to manufacturer specifications, with genomic DNA fragmented to 350 bp. Library preparation and sequencing for the pied avocet was completed at IKMB using one lane of a flow cell on an Illumina HiSeq 4000 for 2 × 150 bp sequencing.

### 2.3. Reference Genome Sequence Processing and Assembly

#### 2.3.1. Kakī and Australian Pied Stilt

Raw kakī and pied stilt sequence reads were evaluated for quality using FastQC v. 0.11.5 [54]. To test for exogenous contamination, the presence and abundance of non-avian reads was estimated by randomly subsampling 5000 reads from each library and searching these reads against the NCBI nucleotide database using BLAST [55].

Illumina adapters used for sequence barcoding were removed using Trimmomatic v. 0.35 [56]. Low quality bases were trimmed using ConDeTri v. 2.3 [57] with default settings. Read deduplication was carried out with ConDeTri, using the first 50 bp of both reads in a pair for comparisons. Raw reads were analysed using SGA-preqc v. 0.9.4 [58] to generate estimates of genome size and heterozygosity. To determine the level of expected heterozygosity in the genome and assess potential signatures of contamination, paired-end reads were analysed using KmerGenie [59].

Trimmed sequences were assembled with SOAPdenovo2 [60] following initial testing of several assemblers and varying k-mer values. Draft assembly metrics were independently assessed with the assembly metrics script generated for Assemblathon [61]. BUSCO v. 3.0.1 [62,63] was used to determine completeness of the assembly outputs based on expected gene content using an avian ortholog set derived from OrthoDB v. 9 [64] and the chicken as reference. Both assembly metrics and BUSCO scores were used to determine the highest quality assemblies.

Trimmed sequence reads were used to close gaps between scaffolds in the highest quality assemblies for kakī and pied stilt with GapCloser v. 1.12 [60]. Scaffolds shorter than 5 kbp were removed, and genomes were syntenically aligned against the chicken reference genome (version 5.0, GenBank Assembly GCF_000002315.5) using Chromosemble in Satsuma v. 3.1.0 [65] to generate pseudochromosome-level assemblies by aligning the draft assembly scaffolds against the chicken genome, and retaining orthologous regions. Final drafts of kakī and pied stilt genomes are available (see Data Availability section).

#### 2.3.2. Pied Avocet

Raw pied avocet sequence reads were evaluated for quality using FastQC v. 0.11.5 [54]. To remove low quality reads, paired-end data was trimmed for Illumina adapter contamination and low quality bases using Skewer v. 0.2.2 [66] with a mean Phred-score of 20, end-trim quality of 30, and a minimum length of 54 bp. Raw reads were analysed with SGA Preqc 0.10.15 [58] and KmerGenie [59] to estimate heterozygosity and potential signatures of contamination. These analyses indicated high expected heterozygosity (0.3%) compared to other birds. To eliminate highly abundant repeats and sequencing errors, a digital normalisation was conducted using Khmer 2.1.1 [67].

Pied avocet trimmed sequences were assembled using Velvet 1.2.10 [68] following initial testing with Meraculous-2D v. 2.2.5.1 [69], which failed to produce a high-quality assembly due to an overabundance of incorrectly merged diplotigs (i.e., contig pairs that share a unique k-mer at both ends [70]). To evaluate the misassemblies, a second assembly was done with Velvet using default parameters. All contigs were aligned against the assembly using LAST [71], with the -uNEAR seeding parameter. Alignments were filtered for trivial self-vs-self perfect alignments, with only single high-scoring pairs per sequence over 99% identical kept. These alignments revealed an unusual number of large and frequent indels (> 3 bp, higher than the default Velvet parameter for allowed gaps in graph bubbles) in extremely similar contigs, and therefore a final Velvet assembly was run with adjusted parameters (-ins_length 410, -max_branch_length 50, -max_divergence 0.1, -max_gap_count 10).

Assembled scaffolds were analysed with GapCloser v. 1.12 [60] to decrease gaps in the assembly. The gap-closed assembly was then aligned against the chicken genome using LAST [71] and the Chromosomer [72] toolkit was used to construct superscaffolds. The final draft of the pied avocet genome is available (see Data Availability section).

#### 2.3.3. Killdeer

A killdeer genome was published in the ordinal phase of the B10K project [21]. To improve the assembly, a full de novo approach was used to construct a low-level base-accurate assembly. The data used in the original assembly of killdeer was downloaded from the GigaDB website [73]. This consisted of 12 libraries of Illumina sequence data, including five paired-end libraries with insert sizes ranging from 170 bp to 800 bp and seven mate-pair libraries of insert sizes ranging from 2000 bp to 20,000 bp.

FastQC v. 0.11.5 [54] was used to evaluate the quality of the Illumina data, as well as assess the contamination levels present in the samples. All paired-end libraries consisted of paired 100 bp reads, whereas mate-pair libraries were constructed of paired 50 bp reads. There was no evidence of any significant DNA contamination, but the per-base Phred-scores showed a consistently lower quality early in the reads. Due to the issues observed in the FastQC reports, reads were trimmed using Skewer v 0.2.2 [66] to a minimum Phred-score of 30 and any read pair where at least one of the mates was trimmed to a length of < 32 bp was discarded.

Trimmed sequences were assembled using AllPaths-LG [74,75] following initial testing of several assemblers and varying k-mer values. The first run was made with the two 170 bp libraries and the complete collection of mate-pair libraries. As part of the AllPaths-LG pipeline, a set of diagnostic data was generated, including estimates of genome size, error rates, and SNP rates. Three of the mate-pair libraries were removed from subsequent analysis after low levels of utilisation were detected due to failed library construction.

The new draft assembly was aligned against the original killdeer reference genome produced by Zhang et al. [21] using the program LAST [71], which identified areas of conflict between the original and new draft killdeer genomes (e.g., short gaps, abundance of small indels, and poor resolution in heterozygous regions in the original genome). A custom set of scripts, ‘SemHelpers’ [76], was written to consolidate the changes detected via the genome-wide alignments into the existing reference genome. The resulting assembly has almost identical metrics when compared to the original assembly [21], given the method used. Post-correction alignments between the final assembly and the original reference genome show identities between 98 and 99%.

Quality of all final draft assemblies was assessed with the Assemblathon metrics script [61] and completeness assessed with BUSCO v. 3.0.1 [62,63] using the avian ortholog set and the chicken as reference. The final draft of the killdeer genome is available (see Data Availability section).

### 2.4. Genotyping-by-Sequencing

Genotyping-by-sequencing (GBS), a reduced-representation genomics approach, was used to produce genome-wide SNPs for kakī. Briefly, GBS reduces genome complexity by sequencing regions that flank restriction enzyme cut sites [77]. The GBS data presented here were produced following the Elshire et al. [77] method, using 50 ng of genomic DNA with 0.72 ng of total adapters and the restriction enzyme ApeKI.

Because the kakī samples were collected during two different breeding seasons, library preparation and sequencing were completed in two separate batches. The first batch included captive parents and offspring from the 2015/2016 breeding season and other individuals sampled from 2014–2015 that represent diverse lineages based on the kakī pedigree (*n* = 52; pedigree data not shown). This batch was sequenced with paired-end, 2 × 100 bp reads on one lane of an Illumina HiSeq 2500 through NZGL. The second batch consisted of captive parents and offspring from the 2016/2017 breeding season plus one wild individual sampled in 2014 who represented a diverse lineage based on the pedigree (*n* = 47). This batch was sequenced with paired-end, 2 × 150 bp reads on one lane of an Illumina X Ten through CustomScience, Ltd. To assess the impact of batch effects (i.e., library and lane biases [78]), 10 individuals were represented in both batches to ensure similar genetic distance estimates were produced by each duplicated sample independently (see Table S1 for individual sample sequencing details).

FastQC v. 0.11.4 [54] was used to evaluate the quality of the raw Illumina data, as well as assess the contamination levels present in the samples. Paired-end reads were demultiplexed and barcodes were trimmed using Axe [79] with a maximum mismatch of 1. To minimise batch effects [78] and address sequence quality, reads from the 2016/2017 breeding season were trimmed to a maximum length of 100 bp using Skewer [66]. To remove low quality data, reads were filtered to discard short reads (< 32 bp) and reads with mean quality scores less than 30.

In order to be read by downstream pipelines, new single-end barcodes were generated for the ApeKI enzyme using the programme GBSX [80] and appended to the forward-end of reads through a custom Perl script, ‘mux_barcodes’ [81]. For this study, the Tassel 5.0 [82] pipeline was used for SNP discovery and genotyping with GBS data. Due to the double-barcoding scheme of the GBS data generated here, a new class of enzymes was created specifically for Tassel 5 to add the enzyme cut site remnant, together with the reverse barcodes, as recognition sites for these datasets. The Tassel 5.0 GBSv2 pipeline was used with tag database and export plugins specifying a k-mer length of 64, a minimum k-mer length of 20, a minimum Phred-score of 30, and a minimum tag count of 10. Bowtie2 [83] was used to align tags to the each draft reference genome using the *--very-sensitive* presetting. The Tassel 5.0 GBSv2 discovery SNP caller plugin was run with a minimum minor allele frequency (-mnMAF) of 0.05 and a minimum locus coverage (-mnLCov) of 0.75. VCFtools v. 1.9 [84] was used to filter the dataset to a set of bi-allelic SNPs, with an average minimum SNP depth of 5, and 90% of all SNPs being shared amongst individuals. To minimise statistical bias of linkage disequilibrium, the data set was pruned for linkage disequilibrium using BCFtools v. 1.9 [85] with r^2^ set to 0.8 and a window size of 1000 sites. To ensure a more even spread of SNPs throughout the genome, VCFTools v. 1.9 [84] was used to reduce the number of SNPs to 1 SNP within 64 bp, which is the designated size of a GBS tag using Tassel 5.0. VCFs of the filtered data set are available (see Data Availability section).

In order to evaluate whether the same SNPs were likely to be mapped using different reference genomes, a custom script, ‘pancompare’ [86], was used to compare pairs of tags in SAM files that are unique or shared between Tassel 5.0 runs using different reference genomes. This method uses tag pair mapping as a proxy for SNP discovery, under the assumptions that tags all start at the restriction cut site and intersecting pairs of tags are likely to discover the same SNPs using different reference genomes.

### 2.5. Resequencing

In addition to a reduced representation approach, we also resequenced kakī genomes from 36 individuals for SNP discovery and genotyping. These individuals include parents and offspring from the 2015/2016 and 2016/2017 breeding seasons (*n* = 24) and other individuals sampled between 2014–2017 that represent diverse lineages based on the pedigree (*n* = 12). Libraries were prepared at IKMB using a TruSeq^®^ Nano DNA Library Prep kit following the manufacturer’s specifications. Libraries were sequenced across 34 lanes on a HiSeq 4000 at the IKMB.

FastQC v. 0.11.4 [54] was used to initially evaluate the quality of the raw Illumina data, as well as assess the contamination levels present in the samples. Reads were trimmed for the Illumina barcode and for a Phred-score of 20 using Trimmomatic [56]. Reads were mapped to each indexed genome using Bowtie2 [83] with the *--very-sensitive* presetting. Resulting SAM files were converted to BAM files and read coverage was analysed using mpileup with Samtools v. 1.9 [85]. To improve the computational efficiency of mpileup, a custom Perl script ‘split_bamfile_tasks.pl’ [87] was created to subdivide BAM files and run them in parallel. SNPs were detected, filtered, and reported using BCFtools v.1.9 [85]. Filtering settings included biallelic SNPs with a minor allele frequency >0.05, an average mean depth >10, and a Phred-score >20. BCFtools was used to filter for a maximum of 10% missing data per site. Resulting SNPs were pruned for linkage disequilibrium using BCFtools with r^2^ set to 0.8 and a window size of 1000 sites. To ensure a more even spread of SNPs throughout the genome, VCFtools v. 1.9 [84] was used to reduce the number of SNPs to 1 marker within 150 bp, which is the length of resequencing reads. VCFs of the filtered data set are available (see Data Availability section).

### 2.6. Diversity Estimates

Nucleotide diversity (*π*) and individual heterozygosity (*H_S_*) were estimated using VCFTools v. 1.9 [84]. Pairwise relatedness (*R*) matrices were produced using KGD [88], a programme that estimates relatedness while taking into account read depth of HTS data. Pairwise *R* values were scaled so that self-relatedness of all individuals was equal to 1 using the formula:

M_S_ = D × M_O_ × D

where *M_S_* is the scaled matrix, *M_O_* is the original matrix, and *D* is a diagonal matrix with elements:

D = 1/√(diag(M_O_)

To compare *H_S_* estimates generated from different reference genomes using GBS and resequencing data, analysis of variance (ANOVA) and Tukey multiple comparisons of means tests were performed using a linear mixed effects model with lme4 [89] to account for repeated measures (i.e., repeated individuals mapped to all four reference genomes). Mantel tests with 1000 iterations were used to test whether scaled pairwise *R* matrices using different reference genomes were significantly similar compared to a null distribution. Correlations were conducted between estimates of *H_S_* and *R* (not including self-relatedness) using different reference genomes using Spearman’s rank (*r_S_*), which accounts for the inherently non-normal distribution of the *R* estimates.

## 3. Results

### 3.1. Reference Genome Sequencing and Assembly

Library sequencing produced 226–307 million paired-end sequences for each kakī, pied stilt, and avocet sample. Average sequencing depth was 52× for kakī, 51× for pied stilt, and 70× for avocet, based on an expected genome size of 1.2 Gb. Genomes produced were between 1.02–1.22 Gb in total length (Table 1), which is within the expected range for avian genomes [90]. Scaffold N50 sizes ranged from 3.66 to 105.71 Mb. The total number of scaffolds ranged from 67 to 15,167. BUSCO assessment indicated the presence of at least 82.4% of the orthologs from the avian database. Combined, these estimates indicate that the assembled genomes have high genome completeness.

### 3.2. SNP Discovery and Diversity Estimates—GBS

After demultiplexing and initial read filtering, kakī GBS sequencing resulted in a total of 802.4 million reads for 88 individuals (mean = 9.1 ± S.D. 4.9 million reads per individual). Five of these individuals were subsequently removed from the study after SNP filtering for having low average sample depths across sites (<4× depth using conspecific reference genome). The resulting 82 individuals have an average depth of 11.71–18.51×, with average missingness of 2–4% depending on the reference genome used (Table 2).

The number of GBS tag pairs mapped to each reference genome was greatest using a conspecific reference genome, with fewer tag pairs mapped the more phylogenetically distant the reference genome became (Table 2). Results from our analysis with ‘pancompare’ [86] indicate that more tags from the congeneric mapping were shared with those mapped to a conspecific reference genome (91.04%) than more distantly related genomes (confamilial = 83.10% and conordinal = 72.42%; Table 2). Tag pairs always start at the GBS restriction enzyme cut site, making direct comparisons of tags mapped across different genomes possible. Because more mapped tags were shared between closely related genomes, these results suggest that SNPs discovered with the congeneric reference genome are more likely the same as those discovered with the conspecific reference genome than those discovered with the confamilial or conordinal references.

**Figure 2:**
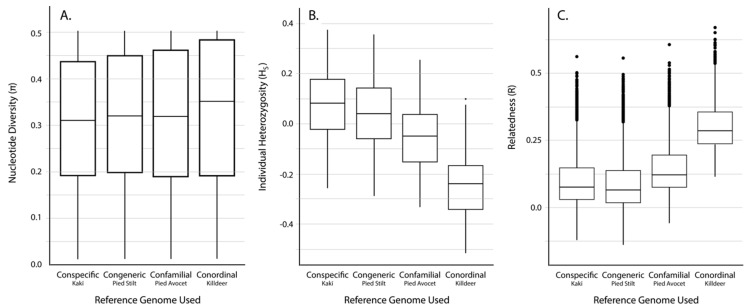
Distribution of different diversity estimates using SNPs discovered with GBS reads mapped against different reference genomes. (**A**) Nucleotide diversity (*π*), (**B**) individual heterozygosity (*H_S_*), and (**C**) pairwise relatedness (*R*) not including self-relatedness.

The number of unfiltered and filtered SNPs discovered was greatest when using a conspecific reference genome, with fewer SNPs discovered the more phylogenetically distant the reference genome became (Table 2). Despite the differences in number of SNPs discovered with each reference genome, average nucleotide diversity (*π*) was similar across datasets (average *π* = 0.31–0.33, Table 2, Figure 2A).

**Figure 3:**
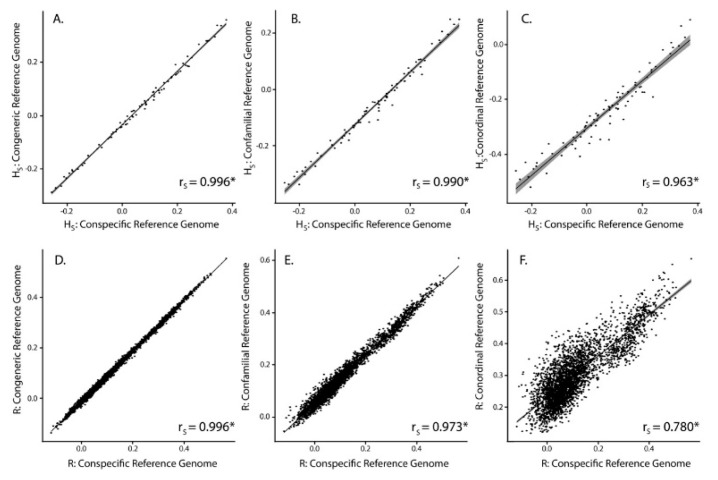
Scatterplots showing individual point estimates of *H_S_* (**A**–**C**) and pairwise *R* estimates (**D**–**F**) using GBS reads mapped to different reference genomes. Self-relatedness estimates were not used in this analysis. Trend line in black, with 95% confidence intervals surrounding the trend line in gray. Spearman’s correlation coefficient (*r_S_*) provided in the lower right corner of each scatterplot. * indicates significance *p* < 0.001.

Average individual heterozygosity (*H_S_*) estimates differed depending on the reference genome used (Table 2, Figure 2B). Results show that using different reference genomes produced significantly different levels of *H_S_* from one another (ANOVA, *p* < 0.001; Tukey Contrasts, *p* < 0.001). Using a congeneric reference genome resulted in *H_S_* estimates that are on average 3.4% less than using a conspecific reference genome, with a confamilial being 12.9% less, and a conordinal being 31.6% less. Despite significant differences in *H_S_* depending on the reference genome used, estimates of *H_S_* using different reference genomes were significantly correlated (Spearman’s correlation, *p* < 0.001), with correlation coefficients between the conspecific and congeneric approaches (*r_S_* = 0.996) being higher than the conspecific and confamilial approaches (*r_S_* = 0.990) and conspecific and conordinal approaches (*r_S_* = 0.963; Figure 3A–C).

The range of scaled average pairwise estimates of relatedness (*R*) shows a bimodal distribution, which reflects highly related individuals (siblings and parent-offspring relationships) along with more distantly related individuals that are captured in the study design. The range of scaled *R* values appeared different depending on the reference genome used, with average pairwise *R* in the conspecific and congeneric analyses being less than the confamilial and conordinal analyses (Table 2). Despite this pattern, the relationships between *R* using a conspecific reference genome and all other genomes were not significantly different (Mantel test, *p* < 0.001). Estimates of pairwise *R* (not including self-relatedness) using different reference genomes were significantly correlated (Spearman’s correlation, *p* < 0.001), with correlation coefficients between the conspecific and congeneric approaches (*r_S_* = 0.996) being higher than the conspecific and confamilial approaches (*r_S_* = 0.973) and the conspecific and conordinal approaches (*r_S_* = 0.780; Figure 3D–F).

### 3.3. SNP Discovery and Diversity Estimates—Resequencing

After demultiplexing and initial read filtering, the kakī resequencing resulted in a total of 4.8 billion reads for 36 individuals (mean = 135.8 ± 54.1 million reads per individual). After SNP filtering, these 36 individuals have an average depth of 13.95–17.44× with average missingness of 0.2% across all reference genomes used (Table 3).

**Figure 4:**
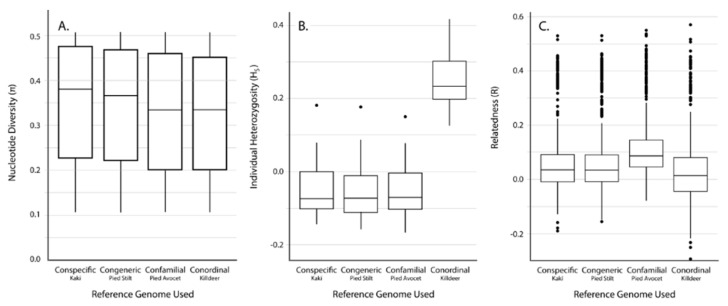
Distribution of different diversity estimates using SNPs discovered with resequencing reads mapped against different reference genomes. (**A**) Nucleotide diversity (*π*), (**B**) individual heterozygosity (*H_S_*), and (**C**) pairwise relatedness (*R*). Self-relatedness estimates were not used in this analysis.

Average read alignment rates using Bowtie2 were highest when using a conspecific reference genome (94.6%), with fewer reads aligning with congeneric (88.1%), confamilial (78.5%), and conordinal reference genomes (64.8%, Table 3). In contrast to GBS, the number of unfiltered SNPs increased with phylogenetic distance of the reference genome, which is expected given resequencing SNPs are called by differences between reads and the reference. The number of SNPs discovered post filtering did not correspond with phylogenetic distance of the reference used, with the fewest filtered SNPs being discovered with the conordinal reference genome (89,145) and the most being discovered with the confamilial reference genome (143,343, Table 3). Similar to the GBS dataset, average *π* was similar across datasets generated using different reference genomes (average *π* = 0.32–0.35, Table 3, Figure 4A).

**Figure 5:**
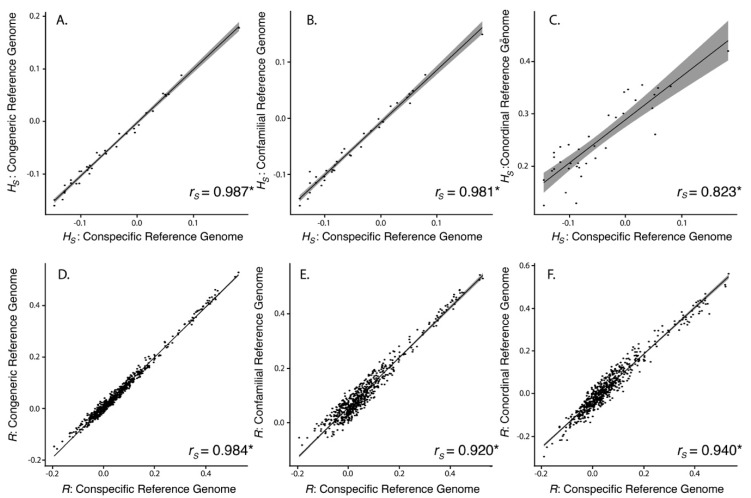
Scatterplots showing individual point estimates of *H_S_* (**A–C**) and pairwise *R* estimates (**D–F**) using resequencing reads mapped to different reference genomes. Self-relatedness estimates were not used in this analysis. Trend line in black, with 95% confidence intervals surrounding the trend line in gray. Spearman’s correlation coefficient (*r_S_*) provided in the lower right corner of each scatterplot. * indicates significance *p* < 0.001.

Results show that using a conordinal reference genome produced significantly higher levels of *H_S_* than the conspecific, congeneric, or confamilial approaches (ANOVA, *p* < 0.001; Tukey contrasts, *p* < 0.001; Table 2, Figure 4B). Using a congeneric reference genome resulted in *H_S_* estimates that are on average 0.40% less than using a conspecific reference genome, with a confamilial being 0.31% less, and a conordinal being 29.9% greater. Despite significant differences in *H_S_* depending on the reference genome used, *H_S_* using different reference genomes is significantly correlated (Spearman’s correlation, *p* < 0.001), with correlation coefficients between the conspecific and congeneric approaches (*r_S_* = 0.987) being higher than congeneric and confamilial approaches (*r_S_* = 0.981) and congeneric and conordinal approaches (*r_S_* = 0.823; Figure 5A–C).

Similar to the GBS approach, the range of scaled average pairwise estimates of relatedness (*R*) based on resequencing also shows a bimodal distribution, which reflects the relationships of individuals captured in the study design. Average scaled pairwise estimates of *R* were similar across all reference genomes used (Table 2, Figure 4C). The relationship between *R* using a conspecific reference genome and all other genomes were not statistically different compared to the null distribution (Mantel test, *p* < 0.001). Scaled pairwise *R* (not including self-relatedness) using different reference genomes is significantly correlated (Spearman’s correlation, *p* < 0.001), with correlation coefficients between the conspecific and congeneric approaches (*r_S_* = 0.984) being higher than conspecific and confamilial approaches (*r_S_* = 0.920) and conspecific and conordinal approaches (*r_S_* = 0.940; Figure 5D–F).

## 4. Discussion

For species of conservation concern, limited conspecific genomic resources often impede inclusion of genomic data in conservation management strategies. Our proof of concept demonstrates that SNPs discovered using congeneric, confamilial, and even conordinal approaches yield diversity estimates that significantly correlate with estimates derived from SNPs discovered using a conspecific approach. Prior to this study, there was only one genome publicly available for the order Charadriiformes (i.e., the killdeer [21]). This study provides three additional high-quality de novo genome assemblies, all of which have practical applications for conservation.

The number of GBS tag pairs that aligned to each reference genome decreased the more phylogenetically distant the reference genome became. Because Tassel 5.0 calls SNPs based on differences among tag pairs [82]—as opposed to differences between tag pairs and the reference genome—the number of unfiltered SNPs discovered also decreased the more phylogenetically distant the reference genome became. The same pattern was observed for the number of filtered SNPs. The ‘pancompare’ analysis of GBS tag data suggests that SNP discovery using the conspecific and congeneric reference genomes are more likely to yield the same markers compared to SNPs discovered using the confamilial or conordinal reference genomes.

The number of resequencing reads that aligned to each reference genome also decreased the more phylogenetically distant the reference genome became. Unlike GBS, the number of unfiltered SNPs increased with phylogenetic distance. This is to be expected because the resequencing discovery pipeline calls SNPs based on differences between reads and the reference genome [85]. The number of SNPs discovered post-filtering was unexpected, however, as a similar number of SNPs were found in all but the confamilial reference approach, which resulted in ~1.5× more SNPs than other reference-guided approaches. While the pied avocet genome shows signs of high completeness, complexities in the genome assembly due to high heterozygosity [69,91] may have resulted in less complete regions leading to higher false discovery rates [41].

Using GBS and resequencing data, the average and range of *π* estimates did not differ greatly based on reference genome used. Larger differences between reference genomes used were observed when estimating *H_S_*. Using GBS data, mean estimates of *H_S_* decreased significantly the more distant the reference genomes became, with the use of a conordinal reference genome producing a marked decrease in *H_S_* estimates compared to the use of a conspecific reference. This decrease in *H_S_* corresponds to an increase in *R*, although not significantly so. These combined results are consistent with expectations because SNPs called by Tassel 5.0 are based on identifying mapped tag pairs [82]; the more phylogenetically distant a reference genome is, the more conserved a region has to be to successfully map a pair of tags. Therefore, with GBS we expect *H_S_* to be lower and *R* to be higher the more phylogenetically distant the reference used is, given that variants at these conserved regions are less frequent.

Using resequencing data, conspecific, congeneric, and confamilial approaches produced *H_S_* that were not significantly different from one another, with the only significant difference seen with the conordinal approach, which resulted in a significant increase in *H_S_* compared to other reference genomes. Unlike GBS tags, there is not an immediate explanation for this pattern. However, it may be attributed to the fact that resequencing reads, which are longer and are more representative of the whole genome, can be mapped to more divergent regions than GBS tags.

While the range of *H_S_* and pairwise *R* values may be different depending on the reference genome used, all estimates produced using different reference genomes correlate significantly with one another. Our results suggest that using a more closely related reference genome (e.g., congeneric) over a more distant reference genome (e.g., conordinal) will yield SNPs that have higher correlation coefficients with estimates generated using a conspecific, and therefore, are likely to result in similar conservation recommendations. Ongoing work incorporating genomic based estimates of relatedness into software that informs captive pairing recommendations (e.g., PMx [92]) will indicate whether more distantly related reference genomes indeed produce statistically similar pairing recommendations, as our correlation results suggest. In the meantime, we anticipate even small changes in *H_S_* and pairwise *R* estimates will not greatly affect conservation recommendations, as diversity estimates are often used in relative terms. For example, pairing recommendations for intensively managed populations that lack reliable pedigrees are routinely informed by genetic- or genomic-based pairwise estimates of relatedness (e.g., [45,93,94,95]). In practice, pairing recommendations are made based on the relative ranking of these estimates and not the absolute values. Similarly, when investigating heterozygosity-fitness correlations (e.g., [96]), relative rankings of *H_S_* among individuals are more informative than absolute values.

Still, there may be some instances where absolute diversity values may be of interest (e.g., parentage assignment, or management of individuals that exhibit *H_S_* below a cutoff score [97]). SNPs derived using the conordinal reference genome provide markedly different ranges of *H_S_* and pairwise *R* estimates and often the lowest correlation coefficients compared to SNPs derived from the conspecific reference genome. For birds, we recommend a confamilial reference genome as the most distant reference genome conservation researchers consider using for diversity estimates. However, this approach should be evaluated for use in other questions, such as the characterisation of adaptive variation [4,14].

The number of de novo bird genomes available to be used as reference is due to increase, especially as the next phase of B10K seeks to publish representative genomes for every recognised family of birds [42]. However, we recommend evaluating the quality of publicly available genomes prior to use, as lower quality genomes may produce lower SNP yield due to fewer alignable regions, or greater false discovery rate where there are assembly errors [98]. Here, we re-assembled the available killdeer reference genome for two reasons. First, the raw data available from the European Bioinformatics Institute European Nucleotide Archive (EBI ENA) showed poor sequencing quality and mapping of this raw data to the existing reference suggested inconsistencies where poor quality reads were more abundant. Second, mapping of the long-insert mate-pair data from the project showed little to no support for many of the scaffolding connections present in the published genome. Due to these factors, we reassembled the genome using much more stringent data curation and more cautious scaffolding. Given this, when using a genome “off the shelf”, we recommend careful assessment of the original genome publication, keeping in mind that genomes assembled from multiple libraries or data types, with greater depth of sequencing coverage, and a more complete and contiguous assembly, will be of higher quality [41]. When genomes with similar phylogenetic relationships are available, comparisons of synteny [65] and completeness [41] against the most closely related model genome may help identify which genome is most appropriate to use. Ultimately, the best way to assess existing genomic resources is to download the raw reads and evaluate them using tools such as FastQC [54] and SGA pre-QC [58], as we have done with the killdeer genome. Raw read quality may have the largest impact on final assembly quality, and initial quality checks will allow identification of any potential anomalies or limitations of the raw data that may have presented challenges to assembly, such as high heterozygosity [69,91,99]. If the raw data is of high quality, but there are inconsistencies between original reported statistics and those derived from raw reads, it may be worth investing in re-assembly to produce a genome of higher quality with greater confidence.

Indeed, re-assembly remains a more cost-effective option than starting a genome sequencing project from scratch. By our current (2018) estimates based on single libraries with paired-end reads, the use of a closely related high quality readily-available reference genome may save a conservation genomic project a minimum of EUR 6500 in library preparations, sequencing, computational power, and assembly time (Table S2, although prices subject to rapid change given new sequencing technologies). Among the 383 species in the order Charadriiformes, 51 are threatened with extinction [100]. The families Laridae (gulls, terns, and skimmers) and Scolopacidae (sandpipers) contain particularly high numbers of threatened species (14 and 13, respectively). Along with the genomes produced in this paper, there are now genomes available for four additional families within Charadriiformes (i.e., Alcidae [90], Charadriidae ([21], here), Recurvirostridae (here), and Scolopacidae [101]). Genome sequencing and assembly of one member of the Laridae family could benefit all 14 threatened species within this family, and combined with the existing genomes available as reference within Scolopacidae, could save conservation groups up to EUR 169,000 in sequencing and assembly costs. Using existing genomic resources will not only reduce these costs, but also the time needed to produce a high-quality reference genome, thereby allowing for a faster uptake of conservation genomics approaches to produce robust information for conservation management.

## 5. Conclusions

Many threatened species management programmes rely on measures of diversity, including nucleotide diversity, heterozygosity, and relatedness, in guiding management decisions [93,102]. While these measures have historically been calculated using small numbers of genetic markers, genomic markers offer the opportunity for increased resolution [1,6,103] and hence improved decision-making. Here, we have demonstrated that in the absence of a conspecific reference genome to map genomic sequence reads to, the availability of high-quality reference genome for a closely related species can provide highly correlated estimates for nucleotide diversity, individual heterozygosity, and relatedness. We anticipate the use of readily available reference genomes may provide resource-constrained conservation projects a way to minimise these costs and make a faster transition to using genomic data to improve conservation outcomes for threatened species.

## Figures and Tables

**Table 1:** Genome assembly metrics for the genomes assembled in this study.

Species	Total Assembly Length (Gb)	Total Scaffolds	Scaffold N50 (bp)	Longest Scaffold (bp)	Average Scaffold Length (bp)	Complete Single-Copy BUSCOs (%)
Kakī	1.18	523	105,710,992	238,324,410	2,254,638	91.0
Pied Stilt	1.12	1443	99,457,149	221,521,436	773,955	85.9
Avocet	1.02	67	87,059,367	184,945,080	15,204,176	82.4
Killdeer	1.22	15,167	3,657,525	21,923,840	80,436	92.5

**Table 2:** Mapping statistics, single nucleotide polymorphisms (SNPs) discovered, SNP descriptive statistics, and average diversity statistics from genotyping-by-sequencing (GBS) reads mapped to different reference genomes. *π*: nucleotide diversity, *H_S_*: individual heterozygosity, *R*: pairwise relatedness (± S.D. for each measure).

Reference Genome	No. of Mapped Tag Pairs	% Tags Shared with Kakī Mapping	No. Unfiltered SNPs	No. Filtered SNPs	Average Missingness	Average Depth	Average *π*	Average *H_S_*	Average *R*
Kaki	392,652	100	634,695	19,396	0.04 ± 0.04	13.73 ± 6.53	0.31 ± 0.14	0.07 ± 0.15	0.11 ± 0.12
Pied Stilt	372,906	91.04	604,573	18,625	0.04 ± 0.04	11.71 ± 5.52	0.32 ± 0.14	0.03 ± 0.15	0.10 ± 0.12
Avocet	316,978	83.10	481,532	18,398	0.03 ± 0.04	13.90 ± 6.58	0.31 ± 0.15	−0.06 ± 0.14	0.15 ± 0.11
Killdeer	151,546	72.42	242,493	10,440	0.02 ± 0.03	18.51 ± 8.77	0.33 ± 0.15	−0.25 ± 0.14	−0.25 ± 0.14	0.30 ± 0.09

**Table 3:** Alignment rates, single nucleotide polymorphisms (SNPs) discovered, SNP descriptive statistics, and average diversity statistics from resequencing reads mapped to different reference genomes. *π*: nucleotide diversity, *H_S_*: individual heterozygosity, *R*: pairwise relatedness. (± S.D. for each measure).

Reference Genome	Average Alignment Rate (%)	No. Unfiltered SNPs	No. Filtered SNPs	Average Missingness	Average Depth	Average *π*	Average *H_S_*	Average *R*
Kaki	94.6 ± 0.50	4,246,100	91,854	0.002 ± 0.005	17.44 ± 6.79	0.35 ± 0.13	−0.05 ± 0.08	0.06 ± 0.11
Pied Stilt	88.1 ± 0.96	8,438,866	89,419	0.002 ± 0.005	14.99 ± 6.06	0.34 ± 0.13	−0.05 ± 0.08	0.06 ± 0.11
Avocet	78.5 ± 0.46	24,333,620	143,343	0.002 ± 0.004	16.02 ± 6.43	0.33 ± 0.14	−0.05 ± 0.07	0.11 ± 0.11
Killdeer	64.8 ± 4.89	62,888,931	89,145	0.002 ± 0.004	13.95 ± 5.54	0.32 ± 0.13	0.25 ± 0.07	0.03 ± 0.13

## Data Availability

The pied stilt Whole Genome Shotgun project has been deposited at DDBJ/ENA/GenBank under the accession RSEF00000000. The version described in this paper is version RSEF01000000. The pied avocet genome raw reads have been deposited in Genbank under project number PRJNA508299. The reassembled killdeer genome is available at http://www.ucconsert.org/data/. Kakī are taonga (treasured) to Māori (the indigenous people of Aotearoa New Zealand), and as such the genomes obtained from taonga species are taonga in their own right. Therefore, the genome for kakī and all VCFs for GBS and resequencing will be made available on recommendation of the iwi (tribes) that affiliate as kaitiaki (guardians) for kakī. A local genome browser is available to view the kakī genome and all VCFs presented here at http://www.ucconsert.org/data/, along with details on how to request access.

## Supplementary Materials

The following are available online at http://www.mdpi.com/2073-4425/10/1/9/s1, Table S1: Samples used in Genotyping-by-Sequencing and resequencing analyses; Table S2: Cost associated with genome sequencing and alignment; lithium chloride extraction protocol.
