# Supplementary material for: Reference Genomes from Distantly Related Species Can Be Used for Discovery of Single Nucleotide Polymorphisms to Inform Conservation Management
Source: Genes (Basel). 2018 Dec 22;10(1):9. doi: 10.3390/genes10010009 (PMC6356778; doi:10.3390/genes10010009)
Supplement: Supplementary file 1 [file genes-10-00009-s001.pdf]

Supplement: *Reference genomes from distantly related species can be used for discovery of Single Nucleotide Polymorphisms to inform conservation management*

Stephanie J. Galla, Natalie J. Forsdick, Liz Brown, Marc P. Hoeppner, Michael Knapp, Richard F. Maloney, Roger Moraga, Anna W. Santure and Tammy E. Steeves

Table S1 -- Samples used in Genotyping-by-Sequencing and resequencing analyses.

Sample ID	Tissue Type	Year Collected	GBS - Batch 1	GBS - Batch 2	Resequencing	Description
240	Blood	2015	✓		✓	Captive Parent
451	Blood	2015	✓		✓	Captive Parent
452	Blood	2015	✓	✓	✓	Captive Parent
453	Blood	2015	✓	✓	✓	Captive Parent
639	Blood	2015	✓	✓	✓	Captive Parent
1360	Blood	2015	✓			Captive Parent
1376	Blood	2015	✓	✓	✓	Captive Parent
1377	Blood	2015	✓	✓	✓	Captive Parent
1409	Blood	2015	✓			Captive Parent
1429	Blood	2015	✓	✓	✓	Captive Parent
1469	Blood	2015	✓	✓	✓	Captive Parent
1565	Blood	2015	✓	✓	✓	Captive Parent
1659	Blood	2015	✓	✓	✓	Captive Parent
1661	Blood	2015	✓	✓	✓	Captive Parent
1736	Blood	2015	✓			Wild Offspring
1738	Blood	2015	✓		✓	Wild Offspring
1744	Blood	2015	✓			Wild Offspring
1762	Blood	2015	✓			Wild Offspring
1763	Blood	2015	✓			Wild Offspring
1764	Blood	2015	✓			Wild Offspring
1860	Blood	2015	✓			Wild Offspring
1864	Blood	2015	✓			Wild Offspring
1872	Blood	2015	✓	✓	✓	Wild Offspring
1892	Blood	2016			✓	Wild Offspring
1903	Blood	2016	✓		✓	Captive Offspring
1904	Blood	2016	✓			Captive Offspring
1905	Blood	2016	✓			Captive Offspring
1906	Blood	2016	✓			Captive Offspring
1907	Blood	2016	✓			Captive Offspring
1908	Blood	2016	✓			Captive Offspring
1915	Blood	2016	✓			Captive Offspring
1920	Blood	2016	✓			Captive Offspring
1921	Blood	2016	✓		✓	Captive Offspring
1931	Blood	2016	✓			Captive Offspring
1932	Blood	2016	✓			Captive Offspring
1934	Blood	2016			✓	Wild Offspring
1936	Blood	2016			✓	Wild Offspring
1944	Blood	2016	✓			Captive Offspring
1947	Blood	2016	✓			Captive Offspring
1948	Blood	2016	✓			Captive Offspring
1949	Blood	2016	✓			Captive Offspring

1950	Blood	2016	✓			Captive Offspring
1952	Blood	2016	✓			Captive Offspring
1953	Blood	2016	✓			Captive Offspring
1957	Blood	2016	✓			Captive Offspring
1964	Blood	2016	✓			Captive Offspring
1966	Blood	2016	✓		✓	Captive Offspring
1969	Blood	2016	✓			Captive Offspring
1970	Blood	2016	✓			Captive Offspring
1971	Blood	2016	✓			Captive Offspring
1975	Blood	2016	✓		✓	Captive Offspring
1976	Blood	2016	✓			Captive Offspring
15_128	Muscle	2016	✓			Captive Offspring
15_129	Muscle	2016	✓			Captive Offspring
15_130	Muscle	2016	✓			Captive Offspring
1980	Blood	2017			✓	Wild Offspring
1993	Blood	2017		✓		Captive Offspring
1994	Blood	2017		✓		Captive Offspring
1995	Blood	2017		✓		Captive Offspring
1996	Blood	2017		✓		Captive Offspring
1998	Blood	2017		✓		Captive Offspring
2000	Blood	2017		✓	✓	Captive Offspring
2004	Blood	2017		✓	✓	Captive Offspring
2012	Blood	2017			✓	Wild Offspring
2013	Blood	2017		✓		Captive Offspring
2014	Blood	2017		✓		Captive Offspring
2015	Blood	2017		✓		Captive Offspring
2016	Blood	2017		✓	✓	Captive Offspring
2023	Blood	2017			✓	Wild Offspring
2028	Blood	2017		✓		Captive Offspring
2029	Blood	2017		✓		Captive Offspring
2030	Blood	2017		✓		Captive Offspring
2032	Blood	2017			✓	Wild Offspring
2034	Blood	2017		✓		Captive Offspring
2035	Blood	2017			✓	Wild Offspring
2050	Blood	2017		✓		Captive Offspring
2054	Blood	2017		✓		Captive Offspring
2057	Blood	2017		✓		Captive Offspring
2058	Blood	2017		✓		Captive Offspring
2059	Blood	2017		✓		Captive Offspring
2074	Blood	2017			✓	Wild Offspring
2078	Blood	2017		✓		Captive Offspring
2081	Blood	2017		✓	✓	Captive Offspring
2091	Blood	2017			✓	Wild Offspring
2110	Blood	2017		✓		Captive Offspring
2111	Blood	2017		✓		Captive Offspring
2112	Blood	2017		✓		Captive Offspring
2115	Blood	2017		✓		Captive Offspring
2118	Blood	2017		✓		Captive Offspring
2119	Blood	2017		✓		Captive Offspring
2120	Blood	2017		✓		Captive Offspring
2121	Blood	2017		✓	✓	Captive Offspring

2122	Blood	2017		✓		Captive Offspring
2123	Blood	2017		✓		Captive Offspring
2124	Blood	2017		✓	✓	Captive Offspring
2125	Blood	2017		✓	✓	Captive Offspring
15_114	Germinal Disk	2017		✓	✓	Captive Offspring
16_05	Germinal Disk	2017		✓		Captive Offspring

Table S2. Cost associated with genome sequencing and alignment. Prices for sequencing from competitive sequencing providers (see https://genohub.com/ngs/). Compute instance and storage costs from competitive New Zealand-based cloud computing services (see https://catalystcloud.nz. Work hours computed bioinformatician analysis time at at €58 per hour.

Genome Type	Genome Description	Sequencing	Compute Instance and Storage	Work hours	TOTAL COST
Basic Genome	1 lane of Illumina sequencing; 5 days of 256 Gb memory instance for initial assembly plus refinement; 2Tb temporary storage; 60 hours of bioinformatician analysis time.	€ 1,750	€ 315	€ 3,500	€ 5,565
Short Read Full Genome	100x coverage pair-end reads and 2 mate-pair libraries; 5 days of 256 Gb memory instance for pair-end assembly and 9 days of 128 Gb instance for multiple scaffolding iterations; 2 Tb temporary storage; 100 hours of bioinformatician analysis time.	€ 9,335	€ 370	€ 5,835	€ 15,540
Multi-tech Genome	100x coverage pair-end reads, multiple mate-pair libraries, and 10x coverage PacBio sequencing; 2 Tb temp storage. 2 days with 6 instances of 32 Gb machines for PacBio error correction and 2 days with 6 instances for gap filling/completion with PacBio; 160h of bioinformatician analysis time.	€ 17,500	€ 875	€ 9,335	€ 27,710
Long-read Genome	50x PacBio sequencing, 1 lane of Illumina sequencing, and 5 flowcells of Oxford Nanopore; 14 days of 256 Gb instance for long read assemblies, followed by 5 days of 256Gb instance for short read assemblies, and 5 days of 128 Gb instance for integration of all three technologies; 3Tb of storage; 180 hours of bioinformatician analysis time.	€ 46,670	€ 1,460	€ 10,500	€ 58,630

S1.1 - *Lithium Chloride DNA Extractions Protocol*

A small piece of dried blood or tissue was isolated for each sample. 250µl of lysis buffer (1.0M tris, 0.5M EDTA, 5.0M NaCl, 10% SDS, 50mg/mL proteinase K, and molecular-grade RNase-free water) was added and the reaction was placed on a 55°C heat block overnight (at least 6 hours). When fully digested, 250µl of 5M LiCl solution was added to the digested blood or tissue. Samples were inverted for 1 minute. In a fume hood, 500µl of 24:1 chloroform:asoamyl alcohol was added to each tube. Samples were inverted for 5 minutes. Samples were then centrifuged at 11,000 RPM for 3 minutes, where samples separate into two phases.

The complete top phase of sample was transferred into a new tube and 250µl of 5M LiCl solution was added. Samples were inverted for 1 minute. In a fume hood, 500µl of 24:1 chloroform:asoamyl alcohol was added to each tube. Samples were mixed by inverting for 2 minutes. Samples were then centrifuged at 11,000 rpm for 1 minute, where samples separate into two phases. 500µl of the top phase was transferred to a new 1.5ml tube, being careful not to touch the intermediate film between the two phases. 1ml of cold (-20°C) absolute ethanol was added to each sample. Samples were inverted gently as DNA precipitates out of solution. Samples were then placed in the -20°C freezer for a minimum of 2 hours. Once out of the freezer, tubes were centrifuged for 10 minutes at 12,000 rpm at -20°C.

The supernate in each sample was poured off into a waste container, being careful not to disturb the pellet. 500µl of 70% ethanol was added to each sample to wash the pellet. The pellet was fished out using a pipette tip and transferred to a clean tube. After the pellet air dried, the DNA was resuspended in 50µL of TE8 buffer. The samples were incubated for 37°C for 40 minutes to allow for improved re-suspension. Samples were then stored at 4°C until used, the permenantly at -20°C.
